# Management of controversial gastroenteropancreatic neuroendocrine tumour clinical situations with somatostatin analogues: results of a Delphi questionnaire panel from the NETPraxis program

**DOI:** 10.1186/s12885-016-2901-1

**Published:** 2016-11-07

**Authors:** Isabel Sevilla, Ángel Segura, Jaume Capdevila, Carlos López, Rocío García-Carbonero, Enrique Grande, On behalf of GETNE (Spanish Group of NeuroEndocrine Tumors)

**Affiliations:** 1 Oncology Unit. Hospital Clínico y Regional de Málaga, Colonia Santa Inés s/n, Málaga, 29010 Spain; 2 Oncology Unit. Hospital Universitario La Fe, Avda. de Fernando Abril Martorell 106, 46026 Valencia, Spain; 3 Oncology Unit. Hospital Vall d’Hebron, Pg de la Vall d’Hebron 119-129, 08035 Barcelona, Spain; 4 Oncology Unit. Hospital Marqués de Valdecilla, Avda. Valdecilla 25, 39008 Santander, Spain; 5 Oncology Unit. Hospital Universitario 12 de Octubre, Avda. de Córdoba s/n, 28041 Madrid, Spain; 6 Oncology Unit. Hospital Universitario Ramón y Cajal, Ctra. de Colmenar Viejo km. 9.100, 28034 Madrid, Spain

**Keywords:** Neuroendocrine tumors, NET, Gastroenteropancreatic NETs, Somatostatin analogue, SSA, Delphi study

## Abstract

**Background:**

There are clinical situations (CS) in which the use of somatostatin analogs (SSAs) in patients with neuroendocrine tumors (NET) is controversial due to lack of evidence. A Delphi study was conducted to develop common treatment guidelines for these CS, based on clinical practice and expert opinion of Spanish oncologists.

**Methods:**

A scientific committee identified 5 CS with a common core (c-c) [non-functioning NET, not susceptible of surgery/locoregional therapy, Ki67 < 10 % (except for CS5: >10 %), ECOG ≤ 2], and controversy regarding use of SSAs, and prepared a Delphi questionnaire of 48 treatment statements. Statements were rated on a 1 (completely disagree) to 9 (completely agree) scale. Responses were grouped by tertiles: 1–3: Disagreement, 4–6: Neutral, 7–9: Agreement. Consensus was reached when the responses of ≥2/3 participants were located in the same tertile as the median value of all reported responses for that statement.

**Results:**

Sixty five (81.2 %) of 80 invited oncologists with experience in the management of NETs answered a first round of the questionnaire and 57 (87.7 %) of those 65 answered a second round (mean age 43.5 years; 53.8 % women; median time of experience 9 years). Consensus was obtained in 42 (36 agreement and 6 disagreement) of the 48 statements (87.5 %). Regarding **CS1** (Enteropancreatic NET, c-c, non-progressive in the last 3–6 months), overall, SSA treatment is recommended (a wait and see approach is anecdotal and reserved for fragile patients or with low tumor load or ki-67 < 2 %); **CS2** (Pancreatic NET, c-c), overall, SSA monotherapy is recommended, except when high tumor load or tumor progression exists, where combination therapy would be considered; **CS3** [Gastroenteropancreatic (GEP)-NET, c-c, in treatment with anti-proliferative dose of SSA and progressing], overall, SSA maintenance is recommended at the time of progression, with or without adding molecular targeted drugs; **CS4** (GEP-NET, c-c, and negative octreoscan®), SSA in monotherapy is only considered in low-risk patients (low tumor load and Ki-67 < 5 %); **CS5** [GEP-NET, c-c (ki67 > 10 %), and positive octreoscan®], monotherapy with SSA is mainly considered in patients with comorbidities.

**Conclusion:**

Several recommendations regarding use of SSAs in controversial NET CS were reached in consensus and might be considered as treatment guideline.

## Background

Neuroendocrine tumors (NETs) are neoplasms that originate from the peripheral neuroendocrine cell system and lungs, and are most frequently located in the gastroenteropancreatic (GEP) system [1]. GEP-NETs may present as hormonally functioning or nonfunctioning tumors and have distinct clinical features based on their site of origin [2]. The age-adjusted incidence of GEP-NETs in the United States was 3.65/100,000/year between 2003 and 2007 [3], while data from Europe (United Kingdom) regarding gastrointestinal NETs showed an age-adjusted incidence of 1.32 and 1.33 in males and females, respectively, between 2000 and 2006 [4]. Overall, the age-adjusted incidence of NETs has increased 3.65-fold in the United States and up to 4.8-fold in the United Kingdom over the past four decades. Regarding life expectancy, the median survival of registered GEP-NETs in Spain was shown to be 12 years (75 % at 5 years) [5].

Somatostatin analogues (SSAs) have been mainly used in functioning tumors to improve the symptoms of carcinoid syndrome or symptoms of other functional NETs [6, 7]; however, their antiproliferative properties are beneficial in both functioning and non-functioning tumors [8–10]. The randomized, double-blind, placebo-controlled PROMID study [11], was the first study reporting an antiproliferative effect of the SSA octreotide long-acting repeatable (LAR) in patients with metastatic G1 midgut NETs, prolonging time to tumor progression as compared to placebo in patients with functionally active and inactive tumors. Recently, the randomized, double-blind, placebo-controlled CLARINET study [12], conducted in patients with advanced grade 1 or 2 (Ki67 < 10 %) NETs originating in the pancreas, midgut or hindgut, or of unknown origin, showed that treatment with the SSA lanreotide significantly prolonged progression-free survival (PFS) compared with placebo, regardless of the hepatic tumor burden. The antitumor effects of SSAs can be direct, via the interaction with somatostatin receptors, or indirect, by a complex mechanism leading to immune system modulation, apoptosis induction, and angiogenesis inhibition [13].

Clinical guidelines can help in the management of NET patients [14–21]. However, there are still clinical situations (CS) in which the use of SSAs is controversial due to lack of evidence. It is recognized that clinical experience and expert opinion could help establish recommendations for the management of these situations and thus, the NETPraxis program was created.

The NETPraxis program aimed to develop common treatment guidance for controversial CS regarding the use of SSAs, based on clinical practice experience and expert opinion of Spanish oncologists.

## Methods

A scientific committee of 6 Spanish oncologists with experience in the management of NETs identified 5 CS with a common core [non-functioning NET, not susceptible of surgery/locoregional therapy, Ki67 < 10 % (except for CS5: Ki67 > 10 %), ECOG ≤ 2], and controversy regarding pharmacologic treatment with SSAs: CS1 (Enteropancreatic NET, common core, non-progressive in the last 3–6 months), *wait and see or SSA*?; CS2 (Pancreatic NET, common core), *initial SSA, molecular targeted drugs (MTD) or chemotherapy?;* CS3 [GEP-NET, common core, in treatment with anti-proliferative dose of SSA and progressing], *maintain SSA?*; CS4 (GEP-NET, common core and negative octreoscan®), *initial SSA?;* CS5 [GEP-NET, common core (ki67 > 10 %), *initial SSA?.*

Supported by related bibliography, these 5 CS were discussed in 13 local meetings among a total of 66 Spanish oncologists, including the members of the scientific committee. Based on the results of the discussions, the scientific committee prepared a Delphi questionnaire of 48 statements regarding treatment with SSAs, divided into blocks for each of the 5 CS (see tables in the Results section for the whole list of statements; the references used to discuss each of the 5 CS in the local meetings are contiguous to the corresponding CS in the tables). The Delphi method is a widely accepted technique for reaching a consensus among a panel of experts [22]. The experts respond anonymously to at least two rounds of a questionnaire and are provided with a summary of all responses after each round [23]. The experts may then revise their earlier responses in light of those by other members.

Eighty Spanish oncologists with proven experience in the treatment of NETs (>5 years), including those participating in the meetings, were invited to answer the first round of Delphi questionnaire. From October to November 2015, 65 (81.2 %) of the 80 oncologists anonymously answered the questionnaire online (mean age: 43.5 ± 7.8 years; women; 53.8 %; median years of experience in NETs [p25-p75]: 9 [6–15]). Participants were asked to rate each statement on a scale from 1 to 9 (1 = “completely disagree”; 9 = “completely agree”). Responses were grouped by tertiles: 1–3: Disagreement, 4–6: Neutral, 7–9: Agreement. Consensus on a statement was reached when the responses of ≥2/3 participants (≥66.6 %) were located in the same tertile as the median value of all the reported responses for that statement.

A second round of the Delphi questionnaire was performed containing only the statements for which consensus had not been reached in the first round and statements with a neutral consensus, along with a summary of the responses for those statements in the first round. Only the 65 oncologists who had responded the questionnaire in the first round were invited to answer the second round of the Delphi. From December 2015 to January 2016, 57 (87.7 %) of the 65 oncologists completed the second round of the questionnaire (mean age: 42.8 ± 7.3 years; women; 56.1 %; median years of experience in NETs [p25-p75]: 9 [6–14.5]).

### Statistics

The median and interquartile range (p25-p75) of the answers to every item of the questionnaire were calculated.

Cronbach’s alpha (Cα) was used to measure the internal consistency of the questionnaire. Cα can range between 0 and 1, from lower to greater reliability, with values above 0.7 considered acceptable [24]. Intra-class correlation coefficient (r_i_) was used to assess inter-rater reliability, which is considered as poor for r_i_ values <0.40, fair for r_i_: 0.40–0.59, good for r_i_: 0.60–0.74, and excellent for r_i_: 0.75–1.0 [25]. The correlation between the two rounds of the questionnaire was measured by the Spearman coefficient (r_s_), which is considered as non-existent when r_s_: 0–0.25, weak when r_s_: 0.26–0.50, moderate to strong when r_s_: 0.51–0.75 and strong to very strong when r_s_: 0.76–1 [26]. The Kappa index (k) was calculated to estimate the qualitative agreement between the rounds having into account the three answer groups (1–3, 4–6 and 7–9). Coherence is poor or inexistent when Kappa index is < 0.20, weak from 0.21 to 0.40, moderate from 0.41 to 0.60, good from 0.61 to 0.80 and very good from 0.81 to 1 [27]. All these values were calculated for the overall questionnaire and for each block. Statistical significance was considered when *p* <0.05.

The variation coefficient (VC) of the questionnaire was calculated for every round, along with the delta or relative increase in the second round above the first (VC_second_-VC_first_/VC_first_). When delta is <10 %, there is no large variability between the rounds, and thus, there is no need for another round.

## Results

The questionnaire had good internal consistency (Cα value >0.7 for the total questionnaire and each of the blocks) and inter-rater reliability (r_i_ value ≥0.7 for the total questionnaire and each of the blocks) (Table 1).

**Table 1:** Internal consistency (Cα) and inter-rater reliability (r_i_) of the Delphi questionnaire

	Cα	*p*-value	r_i_	*p*-value
**TOTAL (48 items)**	**0.821**	<0.001	**0.808**	<0.001
CS1 (9 items)	0.868	<0.001	0.792	<0.001
CS2 (16 items)	0.936	<0.001	0.891	<0.001
CS3 (9 items)	0.764	<0.001	0.722	<0.001
CS4 (6 items)	0.705	<0.001	0.673	<0.001
CS5 (8 items)	0.912	<0.001	0.885	<0.001

Bold data are the total

*CS* Clinical situation, *Cα* Cronbach’s alpha, *r*_*i*_ Intra-class correlation coefficient

Between the two rounds of the questionnaire, quantitative correlation, as assessed by the Spearman coefficient, was acceptable, with values from moderate (r_s_ > 0.5–0.75) to strong (r_s_ > 0.76–1) for the questions that were asked in both rounds (25) and for every block. Qualitative agreement was also acceptable, with kappa values from moderate (k > 0.4–0.6) to good (k > 0.6–0.8) for the total 25 questions and for every block.

The VC in the first round was 0.38 ± 0.09 and in the second 0.41 ± 0.09, which means a delta increase of 7.9 %; i.e. lower than 10 %, and thus, a third round was not necessary.

Overall, consensus was obtained in 42 (36 agreement and 6 disagreement) of the 48 statements (87.5 %) (Tables 2, 3, 4, 5, and 6). In the first round, there was consensus in 23 (47.9 %) statements (21 agreement and 2 disagreement) and non-consensus in 25 (52.1 %). Out of these 25, in the second round, there was consensus in 19 (15 agreement and 4 disagreement) and non-consensus in 6. The results observed in each CS are discussed in the next section.

**Table 2:** Items regarding CS1: Patient with non-functioning enteropancreatic NET, non-susceptible to surgery or to loco-regional treatment, and with Ki-67 < 10 %, ECOG ≤2, NON-PROGRESSIVE in the last 3–6 months: Wait and see vs. SSA treatment? [11, 12, 16, 30, 32, 62]

	Previous round^a^	Median (p25-p75)	Median range	Participants in median range *n* (%)	Result
1. Treatment is initiated with SSAs in most of this type of patients, since available evidence shows that even in patients with stable disease, treatment initiation significantly lengthens the time to progression.		8 (8–9)	7–9	58 (89.2)	C - A
2. In the absence of other risk factors (younger age [<60–65 years], important comorbidities or high Ki67), wait and see may be considered in patients with low tumor load (hepatic ≤25 %).	30.8 %: 4–6	7 (4–8)	7–9	38 (66.7)	C - A
3. In the absence of other risk factors (high Ki67 or extra-hepatic disease), wait and see may be considered in fragile patients (with important comorbidities/elderly [>75 years]).		8 (6–9)	7–9	47 (72.3)	C - A
4. In the absence of other risk factors (younger age [<60–65 years], important comorbidities or extra-hepatic disease) wait and see may be considered in patients with low Ki-67 (<2 %).	32.3 %: 4–6	7 (6–8)	7–9	42 (73.7)	C - A
5. Overall, wait and see is not considered in patients with Ki-67 > 5 %.		8 (7–9)	7–9	50 (76.9)	C - A
6. Tumor localization (pancreatic or non-pancreatic) is not a key criterion when deciding between wait and see or initiate treatment with SSAs.	63.1 %: 7–9	8 (7–8)	7–9	48 (84.2)	C - A
7. Based on available evidence, lanreotide is the SSA of choice in the treatment of patients with NET of pancreatic origin and Ki67 > 2 % and <10 %.		8 (7–9)	7–9	54 (83.1)	C - A
8. Based on available evidence, octreotide is the SSA of choice in the treatment of patients with NET of pancreatic origin and Ki67 > 2 % and <10 %.	40 %: 4–6	2 (2–3)	1–3	45 (78.9)	C - D
9. Based on available evidence, SSAs are the treatment of choice in patients with NET of digestive origin and Ki-67 < 2 %.		8 (7–9)	7–9	53 (81.5)	C - A

*CS* Clinical situation, *SSAs* Somatostatin analogs, *NET* Neuroendocrine tumor, *C* consensus, *NC* non-consensus, *A* Agreement, *D* Disagreement

^a^Only applies to statements with 2 rounds; participant % in median range: median range

**Table 3:**
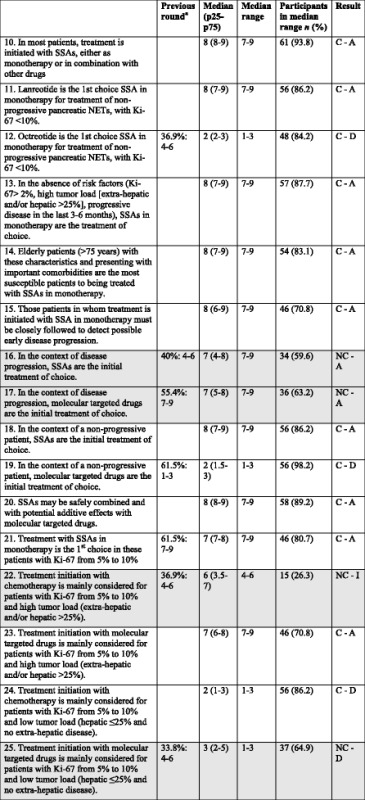
Items regarding CS2: Patient with non-functioning PANCREATIC NET, non-susceptible to surgery or to loco-regional treatment, and with Ki-67 < 10 %, ECOG ≤2: Treatment initiation with SSA, molecular targeted drugs or chemotherapy? [12, 36, 37, 63, 64]

*CS* Clinical situation, *SSAs* Somatostatin analogs, *NET* Neuroendocrine tumor, *C* consensus, *NC* non-consensus, *A* Agreement, *D* Disagreement, *I* Indeterminate

^a^Only applies to statements with 2 rounds; participant % in median range: median range

Shadowed boxes: non-consensus

**Table 4:**
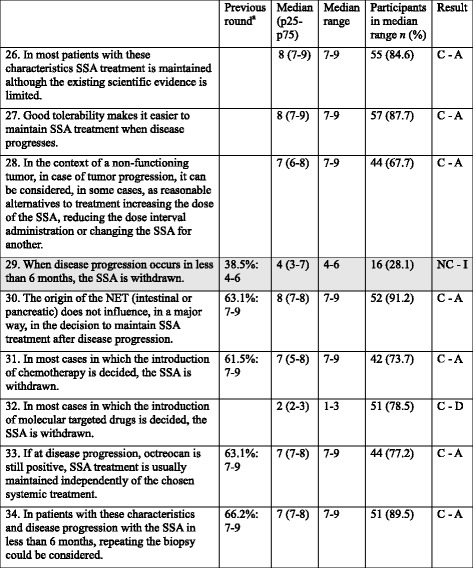
Items regarding CS3: Patient with non-functioning GEP-NET, with Ki-67 < 10 %, ECOG ≤2, in treatment with anti-proliferative dose of SSA and PROGRESSING: Is SSA treatment maintained? [13, 37, 40, 43, 44, 65–67]

*CS* Clinical situation, *SSAs* Somatostatin analogs, *GEP* Gastroenteropancreatic, *NET* Neuroendocrine tumor, *C* consensus, *NC* non-consensus, *A* Agreement, *D* Disagreement, *I* Indeterminate

^a^Only applies to statements with 2 rounds; participant % in median range: median range

Shadowed boxes: non-consensus

**Table 5:**
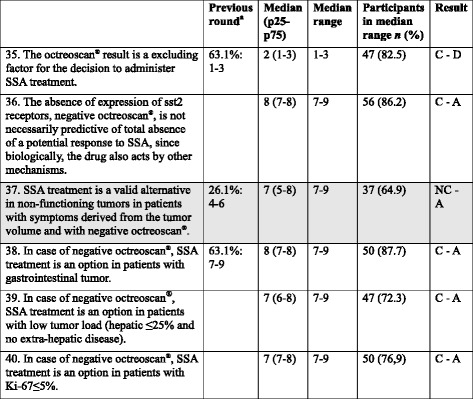
Items regarding CS4: Patient with non-functioning GEP-NET, non-susceptible to surgery or to loco-regional treatment, with Ki-67 < 10 %, ECOG ≤2, AND NEGATIVE OCTREOSCAN®: Is SSA treatment initiated? [11, 48, 49, 68]

*CS* Clinical situation, *SSAs* Somatostatin analogs, *GEP* Gastroenteropancreatic, *NET* Neuroendocrine tumor, *C* consensus, *NC* non-consensus, *A* Agreement, *D* Disagreement, *I* Indeterminate

^a^Only applies to statements with 2 rounds; participant % in median range: median range

Shadowed boxes: non-consensus

**Table 6:** Items regarding CS5: Patient with non-functioning GEP-NET, non-susceptible to surgery or to loco-regional treatment, with Ki-67 > 10 %, ECOG ≤2, AND POSITIVE OCTREOSCAN®: Is SSA treatment initiated? [36, 59]

	Previous round^a^	Median (p25-p75)	Median range	Participants in median range *n* (%)	Result
41. The use of SSA in monotherapy in these patients is reasonable in patients with Ki-67 <20 %.	50.8 %: 7–9	7 (7–8)	7–9	48 (84.2)	C - A
42. The use of SSA in monotherapy in these patients is reasonable in case of low tumor load (hepatic ≤25 % and no extra-hepatic disease).	63.1 %: 7–9	8 (7–8)	7–9	50 (87.7)	C - A
43. The use of SSA in monotherapy in these patients is reasonable in case of NETs of gastrointestinal origin.	52.3 %: 7–9	7 (7–8)	7–9	49 (86)	C - A
44. In case of important comorbidities, SSAs are an option in these patients.		8 (7–9)	7–9	56 (86.2)	C - A
45. In these patients with Ki-67 from 10 to 20 % and/or high tumor load (extra-hepatic and/or hepatic >25 %) and/or NET of pancreatic origin, treatment is usually initiated with chemotherapy.	66.2 %: 7–9	7 (7–8)	7–9	48 (84.2)	C - A
46. In these patients with Ki-67 from 10 to 20 % and/or high tumor load (extra-hepatic and/or hepatic >25 %) and/or NET of pancreatic origin, treatment is usually initiated with molecular targeted drugs (with the possibility of combination with SSA).		7 (6–8)	7–9	45 (69.2)	C - A
47. If discrepancy exists between the degree of cellular proliferation and the octreoscan^®^ results, it is recommended to perform ^18^FDG-PET-TAC to help making a therapeutic decision.	53.8 %: 7–9	7 (7–8)	7–9	46 (80.7)	C - A
48. If discrepancy exists between the degree of cellular proliferation and the octreoscan^®^ results, a re-biopsy of the growing lesions will be considered.	64.4 %: 7–9	7 (7–8)	7–9	53 (93)	C - A

*CS* Clinical situation, *SSAs* Somatostatin analogs, *GEP* Gastroenteropancreatic, *NET* Neuroendocrine tumor, *C* consensus, *NC* non-consensus, *A* Agreement

^a^Only applies to statements with 2 rounds; participant % in median range: median range

## Discussion

A Delphi study was conducted to establish recommendations for the management of CS with undefined protocols regarding the use of SSAs.

CS1 (Patient with non-functioning enteropancreatic NET, non-susceptible to surgery or to loco-regional treatment, and with Ki-67 < 10 %, ECOG ≤2, NON-PROGRESSIVE in the last 3–6 months: Wait and see vs. SSA treatment?)

Due to their proven benefits and tolerable safety profile [11, 12, 28, 29], participants clearly agreed that this type of patients should be treated with SSAs (even in patients with stable disease) rather than considering a wait and see strategy. In the CLARINET study (in which 95 % of the patients had stable disease at baseline) median PFS was not reached in the lanreotide arm vs. 18.0 months (95 % CI: 12.1, 24.0) in the placebo arm [12]. In the extension study, median PFS for patients receiving lanreotide was 32.8 months (95 % CI: 30.9, 68) [29]. Having stable disease before treatment is one of the factors associated with tumor control with lanreotide in patients with well-differentiated malignant digestive NETs [30]. Additionally, a recent *post-hoc* analysis of the CLARINET study has shown that lanreotide was associated with improvements in tumour growth rates (% change in volume per month) vs. placebo as early as 12 weeks (treatment difference: −2.9 [95 % CI: −5.1, −0.8]; *p* = 0.008) [31].

The experts also agreed that SSAs should be the treatment of choice in patients with NET of digestive origin and Ki-67 < 2 %, since almost every patient in the PROMID study and two thirds of those in the CLARINET study had a Ki-67 up to 2 %[11, 12].

However, a wait and see approach could also be considered (although the level of agreement was lower) in patients with low tumor load, elderly patients or with important comorbidities or patients with Ki-67 < 2 % (in absence of other risk factors). No evidence-based data is available with respect to overall survival (OS) to support the early use of SSA vs. wait and see [11, 12, 32], although it is difficult to obtain survival results in patients with slow-growing tumors and long-life expectancy. In any case, participants agreed that wait and see should not be considered if Ki-67 > 5 %.

Currently, there is no clinical data regarding a potential class effect of the two SSAs. Although this issue was not directly addressed in the Delphi questionnaire, the participants agreed with lanreotide, and disagreed with octreotide, being the SSA of choice in the treatment of patients with NET of pancreatic origin and Ki67 > 2 % and <10 % (so far, lanreotide is the only SSA with proven efficacy in pancreatic NETs [12]). In the recent ENETS guidelines update, octreotide is recommended as medical first-line therapy in patients with G1 advanced NETs, originated at midgut, with positive somatostatin receptors and low tumor burden, while lanreotide is recommended as medical first-line therapy in patients with G1/G2 (<10 %) advanced NETs, originated at midgut or pancreas, with positive somatostatin receptors and low or high (>25 %) liver tumor burden [33].

CS2 (Patient with non-functioning PANCREATIC NET, non-susceptible to surgery or to loco-regional treatment, and with Ki-67 < 10 %, ECOG ≤2: Treatment initiation with SSA, MTD or chemotherapy?)

The participants agreed that SSAs monotherapy is the treatment of choice in this patient in absence of high tumor load and in the context of non-progressive disease, mainly due to the ease of use and low adverse effects of these drugs. In this regard, elderly patients with these characteristics and presenting with important comorbidities are considered the most susceptible candidates for SSA monotherapy. Patients receving SSA monotherapy should be closely followed to detect possible early disease progression. The participants agreed that lanreotide should be the SSA of choice in monotherapy in these cases, since it is the only SSA with proven efficacy in pancreatic NETs [33] and patients in the CLARINET study had ki-67 up to 10 % [12].

In the context of disease progression no consensus was reached regarding the initial treatment of choice (although almost 60 % of the participants consider the use of SSA). According to recent ENETS guidelines [33], SSAs may be of value in subgroups of patients with slowly progressive G1 NETs of pancreatic origin, and two prospective randomized trials in metastatic GEP-NETs have shown antiproliferative effects after disease progression [34, 35].

Regarding MTDs, everolimus or sunitinib have shown benefits in two placebo-controlled trials conducted in patients with advanced pancreatic NETs with disease progression [36, 37]. In the study by Raymond et al., patients randomized to sunitinib improved PFS and the objective response rate vs. those receiving placebo [37]. An OS difference favouring sunitinib was observed, but data from the 5 years follow-up was not significant (although crossover might have confounded the results) [38]. In the RADIANT-3 trial, patients randomized to everolimus had longer median PFS than those randomized to placebo [36]. Additionally, the efficacy of everolimus as first-line therapy was confirmed in the subgroup of patients naive to chemotherapy [39].

Consensus agreement was reached on the safe combination and potential additive effects of SSAs with MTDs. This was suggested by the RADIANT-2 study, although it was performed in patients with functioning NETs [40]. In this double-blind, placebo-controlled, phase 3 study comparing octreotide LAR alone with octreotide LAR plus everolimus in patients with advanced, progressive NET with carcinoid symptoms, median PFS were 11.3 and 16.4 months, respectively (*p* = 0.026), failing to reach the level of pre-specified boundary for significance (*p* ≤ 0.0246) [40]. However, “informative censoring” might explain this [41], since when investigators determined that progression had occurred the patient receiving octreotide LAR alone was allowed to cross over to the combination group, but if central review failed to confirm this progression, the patient was censored resulting in inflating PFS values in the octreotide LAR alone group.

Regarding patients with Ki-67 5–10 % and low tumor load, the use of chemotherapy as initial treatment was rejected and a tendency towards rejecting the use of MTDs was also observed. This probably suggests that the use of SSAs in these patients is not uncommon. In patients with Ki-67 5-10 % and high tumor load, the participants agreed on initiating treatment with MTDs.

CS3 (Patient with non-functioning GEP-NET, with Ki-67 < 10 %, ECOG ≤2, in treatment with anti-proliferative dose of SSA and PROGRESSING: Is SSA treatment maintained?)

The participants agreed on maintaining SSA in this patient (mainly due to its good tolerability), independently of whether a MTD is added [42, 43]. Most studies have shown a tendency for improved PFS in the combination arm vs. the single MTD arm, without reaching statistical significance. In the RADIANT-2 study (functioning NETs), patients who received everolimus/octreotide LAR had longer median PFS than those who received octreotide LAR alone, regardless of previous SSA exposure [with previous exposure: PFS 14.3 months (95%CI: 12.0–20.1) vs. 11.1 months (95 % CI: 8.4–14.6); without: 25.2 months (95 % CI: 12.0-not reached) vs. 13.6 months (95 % CI: 8.2–22.7)] [44]. A subanalysis of the Phase III sunitinib study conducted in patients with advanced and progressing pancreatic NETs showed improved PFS in patients receiving SSAs as compared to those who did not, but it was not significantly different [45]. Therefore, there was disagreement with statement 32 about withdrawing SSA when introducing a MTD. In contrast, SSAs are usually withdrawn if chemotherapy is started.

Additionally, the study participants consider increasing the dose of the SSA, reducing the dose administration interval or changing one SSA to the other in these patients.

CS4 (Patient with non-functioning GEP-NET, non-susceptible to surgery or to loco-regional treatment, with Ki-67 < 10 %, ECOG ≤2, AND NEGATIVE OCTREOSCAN®: Is SSA treatment initiated?)

The octreoscan® result was rejected as an excluding factor for the decision to administer SSA treatment, since SSAs do not only exert an effect on hormone secretion but have indirect antiproliferative effects, which do not require the expression of all sybtypes of somatostatine receptors [13, 46, 47]. In addition, the octreoscan® may identify only a specific subtype of somatostatin receptor, such as type 2 [48]. In fact, patients with negative octreoscan® have been shown to respond to SSA [49], and patients with negative octreoscan® were responsive in the PROMID study [11].

According to the results of sentences 39 and 40, monotherapy with SSAs is not considered in patients with negative octreoscan®, high tumor load and Ki67 > 5 %. A retrospective study in patients with digestive NETs treated with lanreotide showed that Ki-67 ≤ 5 % and hepatic tumor load ≤25 % were significantly associated with disease stability [30]. However, it must be taken into account that the CLARINET study showed the efficacy of lanreotide in tumors with Ki-67 up to <10 %, and half of patients had >25 % liver involvement and benefited from treatment [12].

Frequently, an octreoscan® result only considers whether an uptake of the radiolabeled SSA has occurred or not; however, the Krenning scale [lower than (grade 1), equal to (grade 2), or greater than (grade 3) normal liver tissue; or higher than normal spleen or kidney uptake (grade 4)] [50]] suggest that results considered as negative could be positive.

CS5 (Patient with non-functioning GEP-NET, non-susceptible to surgery or to loco-regional treatment, with Ki-67>10 %, ECOG ≤2, AND POSITIVE OCTREOSCAN^®^: Is SSA treatment initiated?)

A higher Ki-67 % is perceived as worse prognosis, and thus, more aggressive treatment is recommended; therefore, in this patient SSA are usually combined with other options.

The participants agreed that when Ki-67 is 10–20 % (i.e., WHO grade 2 [51]), and/or there is high tumor load and/or NET of pancreatic origin, treatment is usually initiated with MTDs (usually combined with SSA) or chemotherapy. In the RADIANT-3 and RADIANT-4 studies, conducted in patients with progressive, advanced, pancreatic (RADIANT-3) or lung or gastrointestinal (RADIANT-4) NETs, including intermediate-grade tumors, everolimus resulted in significantly prolonged PFS vs. placebo [36, 52], and the addition of octreotide to everolimus was previously shown to be well tolerated [42]. Other studies conducted in patients with advanced, pancreatic NETs, including intermediate-grade tumors, have achieved substantial clinical benefits with streptozotocin and 5FU or doxorubicin, capecitabine and temozolomide chemotherapy [53–55] or with oxaliplatin-based chemotherapy [56–58]. In addition, platinum-based chemotherapy resulted in a median survival of 11 months in a study conducted in patients with G3 (Ki-67 > 20 %) gastrointestinal neuroendocrine carcinoma, and survival was significantly better in tumors of pancreatic origin than colon origin [59].

The use of SSA in monotherapy could be considered when Ki67 < 20 %, when there is low tumor load, and in case of important comorbidities, since SSA results mainly in tumor stabilization, and has low toxicity [34, 46, 60]. It also seems reasonable in tumors of gastrointestinal origin, since most of the evidence for chemotherapy and MTDs has been observed in tumors of pancreatic origin (although >50 % of the patients in the everolimus arm in the recent RADIANT-4 study had gastrointestinal NETs [52]).

Additionally, a ^18^FDG-PET-TAC or a re-biopsy can be performed in case of discrepancy between the degree of cellular proliferation and the octreoscan® results.

The study presented the limitations intrinsic to the subjective nature of the answers. In addition, the number of oncologists invited to participate was reduced (given the low frequency of the disease), and thus, the number of questionnaires evaluated was limited. However, the response rate was high in both rounds of the questionnaire (>80 %), and the results reflect clinical practice in the field of NETs in Spain. Only the questions not reaching consensus in the first round, along with those with neutral consensus, were passed on to the second round in order to shorten the time needed to answer the questionnaire, to reduce participant fatigue [61]. The questionnaire showed great internal consistency and high quantitative and qualitative correlation between the two rounds for the questions that needed the second round, and thus, the answers may be considered reliable.

## Conclusion

A series of recommendations based on the clinical practice and opinion of Spanish oncologists with experience in the management of NETs were developed and may be used as treatment guidance for the use of SSAs in controversial NET CS.

## Abbreviations

CS: Clinical situations
Cα: Cronbach’s alpha
ECOG: Eastern Cooperative Oncology Group
GEP: Gastroenteropancreatic
k: Kappa index
MTD: Molecular targeted drugs
NET: Neuroendocrine tumor
r_i_: Intra-class correlation coefficient
r_s_: Spearman coefficient
SSA: Somatostain analogue
VC: Variation coefficient
